# The Subventricular Zone, a Hideout for Adult and Pediatric High-Grade Glioma Stem Cells

**DOI:** 10.3389/fonc.2020.614930

**Published:** 2021-01-26

**Authors:** Arnaud Lombard, Marina Digregorio, Clément Delcamp, Bernard Rogister, Caroline Piette, Natacha Coppieters

**Affiliations:** ^1^ Laboratory of Nervous System Disorders and Therapy, Groupement Interdisciplinaire de Génoprotéomique Appliquée (GIGA)-Neurosciences Research Centre, University of Liège, Liège, Belgium; ^2^ Department of Neurosurgery, CHU of Liège, Liège, Belgium; ^3^ Department of Neurology, CHU of Liège, Liège, Belgium; ^4^ Department of Pediatrics, Division of Hematology-Oncology, CHU of Liège, Liège, Belgium

**Keywords:** glioblastoma, recurrence, subventricular zone, glioma stem cell, cancer stem cell, diffuse intrinsic pontine glioma, high grade glioma, diffuse midline glioma

## Abstract

Both in adult and children, high-grade gliomas (WHO grades III and IV) account for a high proportion of death due to cancer. This poor prognosis is a direct consequence of tumor recurrences occurring within few months despite a multimodal therapy consisting of a surgical resection followed by chemotherapy and radiotherapy. There is increasing evidence that glioma stem cells (GSCs) contribute to tumor recurrences. In fact, GSCs can migrate out of the tumor mass and reach the subventricular zone (SVZ), a neurogenic niche persisting after birth. Once nested in the SVZ, GSCs can escape a surgical intervention and resist to treatments. The present review will define GSCs and describe their similarities with neural stem cells, residents of the SVZ. The architectural organization of the SVZ will be described both for humans and rodents. The migratory routes taken by GSCs to reach the SVZ and the signaling pathways involved in their migration will also be described hereafter. In addition, we will debate the advantages of the microenvironment provided by the SVZ for GSCs and how this could contribute to tumor recurrences. Finally, we will discuss the clinical relevance of the SVZ in adult GBM and pediatric HGG and the therapeutic advantages of targeting that neurogenic region in both clinical situations.

## Introduction

Gliomas are the most frequent primary tumors of the central nervous system, both in adults and children. Among them, glioblastoma (GBM), classified as grade IV by the World Health Organization (WHO), is the most frequent in adults, with an overall average annual age-adjusted incidence rate of 3.2 per 100,000 (1, 2). In children, WHO grades III and IV gliomas are generally grouped together as high grade gliomas (HGGs), and are less common, with an annual age-adjusted incidence rate of 0.08 and 0.15 per 100,000, respectively (1). Diffuse intrinsic pontine glioma (DIPG) is the second most common HGG of childhood (3). This tumor has a diffuse growth pattern and is localized in the brainstem. Tumors with identical characteristics are found in other midline structures such as the thalamus and the spinal cord, reason why it has been re-classified as diffuse midline glioma (DMG) in the last WHO 2016 classification (2). In addition, these are phenotypically and molecularly distinct from the other types of HGGs with the characteristic of having histone H3 mutations (2, 3). However, for the interest of this review, DMGs will be included with HGGs in our discussion.

Despite clear molecular and genetic differences between pediatric and adult HGGs, as reviewed by Sturm (4), there seems to be a continuum between these two age groups. For examples, DMG with H3K27M mutations, initially thought to exclusively occur in children, can also be seen in adults (5, 6), while adult GBM characteristics, such as epidermal growth factor receptor (EGFR) amplification, can be seen in adolescents (7). Furthermore, pediatric and adult HGGs share a poor prognosis due to an almost systematic relapse of the tumor despite a multimodal therapy which classically consists of the tumor resection, whenever possible, followed by radiotherapy plus concomitant and adjuvant temozolomide (TMZ) (2, 8). Recurrences most likely emanate from tumor cells which have infiltrated the parenchyma and are practically impossible to successfully eradicate through a complete surgical resection (9). Moreover, some tumor sites are not reachable by surgery which is classically the case for DMG (3).

In addition to cancer cells which have intruded the tumor surroundings, recurrences can be explained by the existence of cancer stem cells (CSCs) or glioma stem cells (GSCs). CSCs-based tumor initiation, growth and maintenance was first proposed over 150 years ago by Virchow, who first suggested that a quiescent sub-population of embryonic stem cells was able to generate tumors (10). Since then, the existence of CSCs in tumors has been demonstrated in various types of cancers (11–13), including in adult GBM (14–17) and pediatric HGG (18–20). Several GSC features supports the hypothesis that these cells contribute to recurrences (16). Indeed, GSCs adapt and survive environmental stresses, present increased resistance to standard therapies and are able to form a novel tumor (21–27). GSCs are mainly present in the tumor mass but have also been detected in the subventricular zone (SVZ), a neurogenic niche persisting after birth and containing resident neural stem cells (NSCs) (19, 21, 23).

The present review will describe the similarities between GSCs and NSCs. The migratory routes of GSCs from the tumor mass to the SVZ and the signaling pathways involved in their migration will also be described hereafter. In addition, we will debate the advantages of the microenvironment provided by the SVZ for GSCs and how this could contribute to tumor recurrences. Finally, we will discuss the clinical relevance of the SVZ in adult GBM and pediatric HGGs and the therapeutic advantages of targeting the SVZ in both clinical situations.

## Glioma Stem Cells Share Features With Neural Stem Cells

Gimple and colleagues recently suggested the following definition for GSCs; “GSCs are defined by tumor-initiating capacity following serial transplantation, self-renewal, and the ability to recapitulate tumor heterogeneity” (16). These functional characteristics are currently the only tools available for the identification of GSCs as none of the stem marker expression shows sufficient sensitivity and specificity. Indeed, GSCs markers widely overlap with NSC specific ones, thus rendering the identification of GSCs within a heterogeneous tumor rather difficult. Thus, so far, GSCs are still solely recognized based on their functional properties (11, 15).

Multiple signaling pathways involved in normal NSC biology also play a role in GSCs. For example, the Notch pathway is implicated in the maintenance of NSCs in an undifferentiated state *via* the repression of proneural gene expression and is frequently upregulated in GSCs (28, 29). The Bone Morphogenetic Protein pathway inhibits neurogenesis and promotes gliogenesis in NSCs, while it stimulates astrocyte-like differentiation and reduces proliferation in GSCs (30, 31). The Wnt pathway regulates NSC and GSC proliferation *via* the accumulation of β-catenin (32, 33). The Sonic Hedgehog pathway is involved in self-renewal of NSCs and GSCs *via* Gli1 (34, 35). STAT3 is needed for NSC and GSC proliferation and the maintenance of multipotency (36, 37). Finally, EGFR, classically expressed by NSCs and promoting their proliferation, is often overexpressed in GBM and has been associated with tumor initiation, tumor growth, cell invasion, angiogenesis, and resistance to chemo- and radiotherapy (38).

In the same way, multiple transcription factors are common between NSCs and GSCs. Bmi1, a component of the Polycomb Repressive Complex 1, is classically found in undifferentiated NSCs and is involved in the maintenance of their multipotency. It also contributes to glioma aggressiveness *via* NF-κB and matrix metalloproteinase-9 (39). In addition, the inhibition of c-Myc, a transcription factor involved in the regulation of stem cell renewal and proliferation, triggers GSC apoptosis and reduces neurospheres formation (40). Sox2 which protects NSCs from apoptosis *via* survivin overexpression (41), is essential for the stemness maintenance of GSCs, together with Oct4 and Nanog (18). Finally, Olig2, a key transcriptional factor normally required for neural progenitor cell (NPC) proliferation (42), is able to reduce the suppressive action of p53 which regulates the proliferation in GSCs (43).

The identification of markers permitting the detection of GSCs is important since it has been estimated that approximately only one GSC every 1,000 tumor cells is present in a GBM tumor (44). Despite this low number of GSCs in the tumor mass, there is now evidence that these cells might contribute to tumor recurrences. Indeed, GSCs can leave the tumor mass, invade the parenchyma and migrate to further locations, including the neurogenic zones, where they escape a surgical intervention (23, 45–47). These GSCs can then remain quiescent until a still unknown mechanism triggers the development of a new tumor (48).

### The Subventricular Zone, a Hideout for Glioma Stem Cells

#### The Architectural Organization of the Subventricular Zone

In the adult human brain, there are two well-described neurogenic zones: the SVZ, situated in the walls of the lateral ventricles (LV), and the subgranular zone (SGZ) of the hippocampal dentate gyrus. Although controverted, some evidence suggest that the presence of NSCs is not limited to these two well-known neurogenic niches but could extend to other parts of the human central nervous system (49, 50). In addition, multiple other niches of NSCs have been reported in other mammalians (51). As the SVZ is the largest neurogenic region of the adult brain and because a link between the SGZ and brain tumors is less clear (52), this review will focus on the role of the SVZ in HGG recurrences.

**Figure 1:**
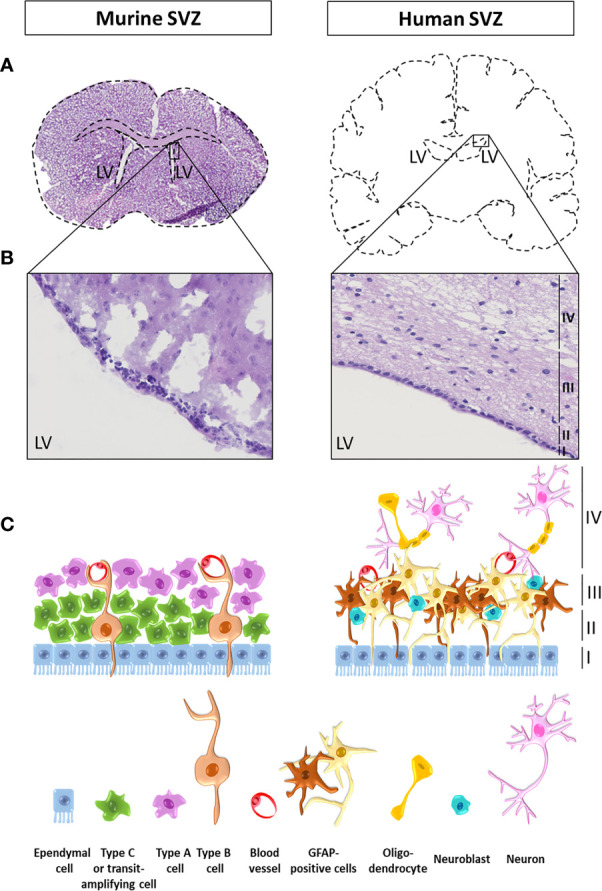
Illustration and schematic representation of a mouse and a human subventricular zone. **(A)** Coronal sections of a mouse (left) and a human (right) brain at the level of the lateral ventricles (LV). **(B)** Zoomed images of a mouse and a human brain subventricular zone stained with haematoxylin and eosin. The four human layers are indicated as layers I to IV from the lumen of the LV toward the parenchyma. **(C)** Schematic representation of the cellular composition of the mouse and the human subventricular zone (SVZ). In adult rodents, neural stem cells (NSCs), also called type B cells, are separated from the LV lumen by ependymal cells. Type B cells undergo asymmetrical cell divisions to give rise to a new type of B cell population with self-renewal properties (one of the hallmarks of a stem cell), as well as transit amplifying progenitor cells, also known as type C cells. Type C cells then migrate to become neural precursor cells including migrating neuroblasts (type A cells) or oligodendrocytes precursor cells. In the SVZ, type B cells display a double contact, one with the ventricle and one with the basal lamina of blood vessels, where the blood-brain barrier (BBB) is not complete. The human SVZ is composed of four distinct layers going from I to IV from the innermost toward the outermost layer. Layer I runs alongside the ventricular cavity and is a monolayer of ependymal cells responsible for the production and secretion of cerebrospinal fluid. Layer II is known as the hypocellular space as it contains cellular processes with only very few cell bodies. Layer III is a cellular ribbon mainly comprising cells expressing glial fibrillary acidic protein (GFAP) and neuroblasts. Finally, layer IV, the outermost layer adjacent to the parenchyma, is a transition zone mainly composed of myelinated axons and oligodendrocytes.

The human SVZ is composed of four distinct layers going from I to IV from the innermost toward the outermost layer. Layer I runs alongside the ventricular cavity and is a monolayer of ependymal cells responsible for the production and secretion of cerebrospinal fluid. Layer II is known as the hypocellular space as it contains cellular processes with only very few cell bodies. Layer III is a cellular ribbon mainly comprising cells expressing glial fibrillary acidic protein (GFAP). Finally, layer IV, the outermost layer adjacent to the parenchyma, is a transition zone mainly composed of myelinated axons and oligodendrocytes (**Figure 1**) (53). Describing the exact localization of NSCs within the different layers of the SVZ is difficult as it depends on which criteria have been used to define or identify these cells. Recent single-cell RNA sequencing studies helped classifying NSCs into four main populations: quiescent NSCs, activated NSCs, NPCs and neuroblasts (54). Most NSC seem to be quiescent and positive for GFAP and CD133 (55). Mammalian NSCs resembling glial cells and sharing common characteristics and markers including GFAP, are mainly detected in layer III of the SVZ (56). The transcription factor Sox2 has been validated for the detection of NSCs in the human fetal brain (57). Sox2 is expressed by quiescent and activated NSCs, but not by NPCs (57, 58). In adults, Sox2 can be detected in the different layers of the SVZ with decreasing numbers toward the parenchyma indicating the presence of NSC in the adult SVZ (59). However, when considering the expression of the immature neuronal markers such as doublecortin, only rare NSCs are found in the adult human brain and only in layer III (60). Finally, in the SVZ, the proliferative marker Ki67 decreases during aging (61) with a limited number of Ki67 positive cells detected in layer III in the adult SVZ, reflecting very few cycling cells (60). However, it is to note that whereas proliferative cells in juvenile SVZ correspond to different cell types including immature cells, in the adult SVZ, Ki67 is exclusively expressed by microglia (60), the primary resident immune cells of the brain (62).

Differences in the cellular composition exist between the SVZ found in children and the adult counterpart, with more proliferative cells in children, indicating higher neurogenesis under the age of four (60, 63). It is also worth to note that in addition to a continuum between children and adult HGGs, there is a progression in the cellular and molecular properties of NSCs hosted in the SVZ from the embryo, through childhood to adulthood (64).

Variations also exist in the organization of the different SVZ layers between species. In adult rodents, NSCs, also called type B1 cells, are separated from the LV lumen by ependymal cells. Type B1 cells undergo asymmetrical cell divisions to give rise to a new type of B1 cell population with self-renewal properties (one of the hallmarks of a stem cell), as well as transit amplifying progenitor cells, also known as type C cells. Type C cells then migrate to become neural precursor cells including migrating neuroblasts (type A cells) or oligodendrocytes precursor cells. In the SVZ, type B cells display a double contact, one with the ventricle and one with the basal lamina of blood vessels, where the blood-brain barrier (BBB) is not complete. B cells are able to form C cells, which in turn divide in A cells that finally migrate and integrate in the olfactory bulb in mice (**Figure 1**). Note that, in human, the SVZ present a dense layer of B cells while there are just a few of A and C cells. For a complete review on neural stem cells in the adult mammalian brain and a good schematic representation of the SVZ in rodent, we would refer readers to the article from Obernier and Alvarez-Buylla (64, 65).

#### Glioma Stem Cells Take Different Routes to Reach the Subventricular Zone

**Figure 2:**
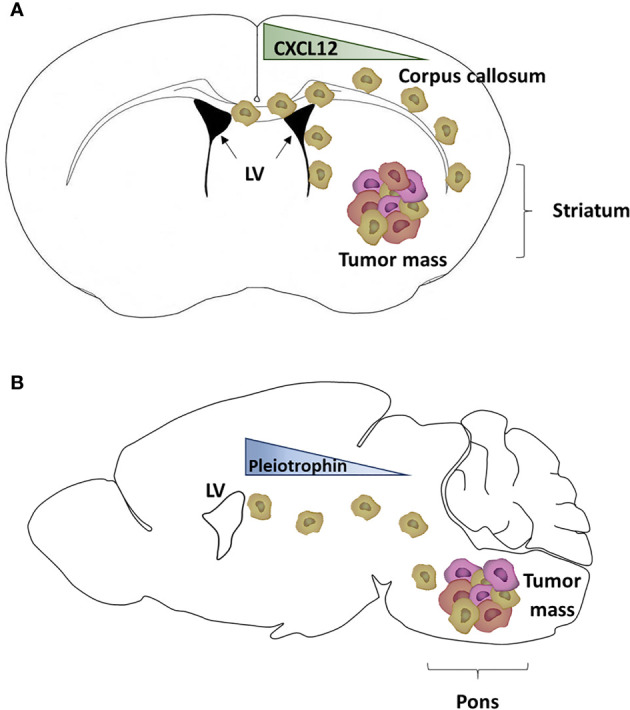
Mouse model of glioblastoma cell invasion into the subventricular zone. **(A)** Schematic representation of adult glioblastoma (GBM) cells grafted into the right striatum of a mouse brain (schematically drawn as a coronal section ahead of the hippocampus) and generating a tumor mass. Some GBM cells expressing CXCR4 (light brown cells) migrated through the corpus callosum to reach the subventricular zone (SVZ) of the lateral ventricles (LV), following a CXCL12 gradient (45, 46). **(B)** Schematic representation of pediatric diffuse midline glioma (DMG) cells grafted into the pons of a mouse brain (schematically drawn as a sagittal section) and generating a tumor mass. Some DMG cells (light brown cells) have migrated out of the tumor mass and reached the SVZ, following a gradient of proteins including pleiotrophin (47).

In non-tumoral brains, white matter tracts can act as a guide, or a motorway, for the migration of NSCs or glial progenitor cells. Multiple evidence highlights a similar pattern of migration for GBM cells. The first evidence of GBM invasion through the white matter tracts was formulated by Scherer and collaborators in 1938 after they studied 100 patients with gliomas. They also demonstrated GBM cell migration through other routes like blood vessels, the neural parenchyma and the subarachnoid space (66). Later, NSCs were transformed to gain tumorigenic capacities before being implanted in mouse brains to model GBM tumor growth and brain invasion. Grafted mice successfully recapitulated GBM tumor development four weeks post-injection. More importantly, two weeks after injection, few GBM cells were detected in the corpus callosum, consisting of white matter tracts connecting the two cerebral hemispheres (67). As reported by our team, the injection of human GSCs in the mouse striatum led to the formation of a tumor. Furthermore, some GSCs left the tumor mass and migrated through the corpus callosum to reach the ipsi- and controlateral SVZ (**Figure 2**). GSCs were also detected in the olfactory bulb, demonstrating their migration from the SVZ through the rostral migratory stream (45), a structure containing a high density of parallel blood vessels classically used as a scaffold for neuroblasts (68). Another interesting study by Kakita et al. revealed the migration of labeled glial progenitors from the neonatal SVZ through the corpus callosum to the contralateral hemispheres, which correlates with the pattern of migration described by Kroonen et al. (45, 69). More recently, diffusion and magnetic resonance performed on seven glioma and six control patients showed that the human corpus callosum also act as a GBM cell migration track (70). In children, Caretti et al. analyzed a series of autopsies from 16 patients with DMG and observed that tumoral cells spread to the SVZ in 63% of the cases (19). For the last ten years, neurogenic niches have received more and more attention as not only it is the largest site for stem cells persisting in adulthood, but also, as discussed above, it can be a preferred destination site for GSCs (23, 45–47).

#### Chemoattractants Secreted by the Subventricular Zone Contribute to Glioma Cell Migration

The SVZ secretes various factors including chemokines and other proteins regulating cell migration (71). Some of the pathways involved in the migration pattern of GSCs from the tumor mass toward the SVZ have already been identified (46, 47).

The first axis, CXCL12/CXCR4, has been identified by our team in 2015 (46). CXCL12 is a chemokine acting on two main receptors, CXCR4 and CXCR7 (72–75). We have demonstrated that CXCL12 is a key player in the migration of CXCR4 positive adult GSCs from the tumor mass toward the SVZ (21). In addition to its chemokine activity, CXCL12 is involved in many biological activities (76) including the regulation of cell proliferation and tumor growth (75), favors an epithelio-mesenchymal transition, regulates the expression of GSC cell markers (77), and increases resistance to both radiotherapy and chemotherapy (21, 22, 78, 79). Furthermore, CXCL12 increases cell survival and facilitates DNA double strands break repairs through the recruitment and phosphorylation of nuclear MAP kinase phosphatase 1. It is also interesting to note that both effects induced by CXCL12 (oriented migration and DNA repair) are dependent on a CXCR4 signalization (78). A potential role for CXCR7 in the mediation of CXCL12 effects on GSCs remains to be investigated since these cells also express that receptor (76, 78).

A second migratory axis has been identified by Qin et al. (47) who showed that pleiotrophin, along with three required binding partners (secreted protein acidic and rich in cysteine (SPARC), SPARC-like protein 1 and heat shock protein 90B) is secreted by SVZ NPC and triggers the migration of DMG and adult GBM cells to the SVZ, through activation of the Rho/ROCK pathway (47).

Additional chemokines are known to be expressed in the SVZ-environment and could also contribute to the recruitment of GSCs (21, 46, 47). Using an array, we studied mouse SVZ conditioned media which led to the identification of several chemokines, including CXCL12 already described above. Amongst the other chemokines, we detected CXCL1, CCL5, CXCL10, and CXCL2. Furthermore, a gene expression profiling analysis was performed using real-time PCR Arrays on total RNA extract obtained from microdissected mouse SVZs. Genes were classified into high, basal and low mRNA levels. Amongst 14 genes highly expressed in the SVZ, we identified CXCL12 as well as CX3CL1 (also known as fractalkine), CCL19 and CCL12 which is the homologue of Monocyte Chemoattractant Protein-1 (MCP1/CCL2) in human (46). In a later study, chemokines were detected in condition media obtained from a human SVZ (21). This technical approach allowed the identification of eight chemokines in the conditioned media, within which Macrophage Inflammatory Protein-3 Alpha (MIP-3α/CCL20) was detected at the highest level, followed by interleukin 8 IL-8/CXCL8, MIP-1α/CCL3, Neutrophil-activating peptide 2 (NAP-2/CXCL7), and MCP-1. Interestingly, at least three chemokines detected in the conditioned media obtained from mouse SVZ were also detected in the human one, namely MCP1, Tazarotene-induced gene 2 protein (TIG2/chemerin) and IL16 (21, 46).

To the best of our knowledge, studies from our team were the only one that employed mouse and human SVZ-conditioned media to identify chemokines released by the SVZ. However, the identification of previously described chemokines was based on targeted techniques. Beside our work, numerous transcriptomic and proteomic studies of the SVZ have been performed; however, without specific focus on SVZ-secreted proteins. Indeed, these studies were based on isolated cells from the SVZ or whole SVZ extracts (61, 80–84). Recently, whole proteins forming the extracellular matrix and their associated proteins, respectively named the “matrisome” and “matrisome-associated proteins”, were extracted from 8 to 12-week-old murine SVZ. In the later study, S100 proteins and Serpins were identified as highly soluble in the SVZ-matrisome. An in-depth analysis of the identified SVZ-associated soluble proteins could potentially lead to the discovery of new migratory-related soluble factors (80). In humans, the composition of the SVZ has mainly been studied during development and is based on the characterization of proteins and/or mRNA expression in specific cell-types and/or on whole SVZs (85, 86). Moreover, databases are now available to study mRNA expression in human age-related SVZ: i.e., BrainSpan Atlas of the Developing Human Brain (83, 87). These databases deserve attention as they could help identify potential SVZ-chemokines. In 2016, the comparison of the secretome of human NSC and GSC cell lines, identified 138 proteins differentially expressed (86). Although this analysis was based on cell lines, the identification of NSC-secreted proteins could help interpret and/or validate future large-scale studies based on SVZ-secreted proteins. Indeed, after reviewing the current literature, it is clear that large-scale analysis of human SVZ-chemokines or secreted proteins are still required.

To conclude, various chemokines are expressed and tightly regulated in the SVZ environment. Numerous studies on human SVZ-secreted proteins would highlight new migratory-related factors and/or confirm the one shown in murine SVZ. Interestingly, some of these secreted proteins would be responsible for specific GSCs migration toward this neurogenic niche. Delocalization of GSCs would be responsible for their maintenance even after tumor resection and their role in HGG recurrences. The identification of specific chemokines, the analyses of their role in GSCs migration capacity and the study of their targeting is therefore of interest to better understand and fight HGG recurrences.

#### The Subventricular Zone Offers Interesting Advantages for Glioma Stem Cells

The association of GSCs with non-tumoral cells together with soluble factors provides specific intra-tumoral microenvironments known as niches. The niche concept can be described as an environment able to maintain NSC stemness (88). This concept has been transposed to gliomas with GSC maintenance, division and differentiation in specific GBM localisations. Whereas perivascular, perinecrotic and invasive niches clearly exist in GBM, these structures are dynamic and are not always easily distinguishable one from another (89). In addition to GBM stem cells, the cellular components of these tumors include lymphocytes, macrophages, fibroblasts, pericytes, astrocytes, microglial cells, and neurons. GBM heterogeneity also occurs in different part of the tumor mass with niches not clearly distinct from each other. Niches tightly regulate GBM pathogenesis such as GBM cell survival, invasion, immune escape, and metabolic needs as well as stemness maintenance. A hypoxic environment can give rise to necrotic areas surrounded by hypoxic palisading GBM and immune cells. GBM cells can also hijack abnormal blood vessels to constitute an angiogenic niche or use blood vessels to invade surrounding brain parenchyma in the invasive niche.

This section of the review will describe the SVZ microenvironment and give an overview of the benefits that the SVZ can provide for GSCs. As going into details for each of these advantages is beyond the scope of this article, we will provide general information on the subject and briefly discuss some aspects of the SVZ environment that is beneficial for SVZ-nested GSCs.

The SVZ encompasses various cell types involved in the maintenance of endogenous NSCs and provides all soluble factors, nutrients and oxygen required for the regulation of their biological processes (90). Even the composition of the extracellular matrix of the SVZ plays a major role in the regulation of neurogenesis, cell proliferation and migration (80). Thus, this brain region is an interesting niche for GSCs as by providing an environment adapted for NSCs it also gives the same advantages to SVZ-nested GSCs. The interaction of GSCs with the tumor microenvironment is in fact key for the maintenance of their malignancy (91). In addition to the different cell types composing the SVZ (see above), neurogenic niches encompass other cell types able to secrete soluble factors which can directly act on GSCs and regulate major biological processes involved in the development of the pathology. These cells include microglia, NPCs, and cells composing the architecture of a large vasculature network with specialized properties (91). Indeed, the BBB in the SVZ consists of a vasculature lacking astrocyte end-feet and pericyte coverage at sites. In GBM, those specialized blood vessels are altered which leads to the dysregulation of numerous factors in the brain (92, 93).

NSCs from the SVZ present an increased resistance to TMZ and radiation therapy (21, 23). This radioresistance can be explained in part by high expression of the anti-apoptotic proteins Bcl2 and Mcl1 (94). Interestingly, GSCs nested in the SVZ differ from those which remained in the tumor mass, with SVZ-nested GSCs presenting an increased resistance to irradiation (21). This increased resistance is at least partially regulated by the presence of high levels of CXCL12 in the SVZ (95). Indeed, our group has demonstrated that in addition to attracting GSCs in the SVZ, CXCL12 had a protective effect against irradiation (21). The addition of SVZ-conditioned media to human GBM cells led to a decrease in histone variant H2AX phosphorylation on Ser-139 (γH2AX), a reliable molecular marker of DNA damage repair (21). One of the mechanism involved, is the recruitment and the phosphorylation of MKP1, regulated by CXCL12, which in turn regulates DNA strand breaks repair (78). A radioprotective effect of CXCL12 is supported by a recent study by Rajendiran et al. showing that the ubiquitous overexpression of CXCL12 in a mouse model led to a significant increase in the number of multipotent progenitors and increased radioresistance by promoting quiescence (96). Piccirillo et al. studied human GSCs isolated from the tumor mass or the SVZ and found that most GSCs, isolated from different patients, were resistant to TMZ no matter their origin. GSCs were also resistant to cisplatin, an agent previously used for the treatment of GBM (23).

It is evident that multiple other soluble factors present in the SVZ could promote GSC survival. For example, CX3CL1 highly expressed in the adult SVZ (46) promotes NPC survival (97). Interestingly, CXC3L1 and its receptor are both increased in high grade gliomas, with higher CXC3CL1 being associated with shorter overall survival (OS) (98). CXCL1, also secreted by the SVZ, is overexpressed in GBM tumors, provides radioresistance and is associated with a poorer prognosis for patients affected by the disease (99).

Soluble factors secreted in the SVZ also tightly regulate the balance of NSCs between quiescence and proliferation. These extrinsic signals act through the presence of receptor at the surface of NSCs (100). Amongst receptors enriched in quiescent NSCs, there is for example cadherin 2 which has recently been suggested as a biomarker for the prognosis of GBM and as a predictive factor for the response of gliomas to TMZ (101). In addition to soluble cytokines, oxygen, and nutrients can influence the biology of NSC and thus, of GSCs. The importance of oxygen concentrations for the maintenance of stem cell normal physiology has already been reviewed (102). The level of oxygen found in the SVZ and the SGZ is higher than in the other parts of the brain including the cortex and the thalamus (103). This is interesting since higher oxygen level would help maintain NSC, as well as GSC which have reached the SVZ, in a quiescent and undifferentiated states (104). NSCs in the SVZ are in close contact with the BBB which constantly expose them to circulating molecules and nutrients (105). As already mentioned above, the BBB is often altered in GBM brains (92), which could lead to blood vessel leakage and nutrients unbalance in the SVZ and consequently influence GSC quiescence state (105).

## The Subventricular Zone in Clinical Practice

### An Independent Prognostic Factor

More than 10 years ago, a retrospective clinical study described that a GBM directly in contact with the SVZ at diagnosis was associated with invasiveness and multifocal disease in adults (106). The authors also described four patterns allowing a better characterization of the SVZ involvement: SVZ+/Cortex+ (I), SVZ+/Cortex- (II), SVZ-/Cortex+ (III), SVZ-/Cortex- (IV). A first observational study of 69 patients reported a poorer OS in patients with GBM contacting the LV in comparison to patients with GBM not bordering the LV (107). Multiple studies then confirmed that the SVZ involvement at diagnosis or at recurrence was associated with poorer OS (108–110). Recently, Mistry et al. confirmed in a meta-analysis of fifteen studies and in a retrospective study of 207 adult patients, that GBM contact with the LV was associated with lower OS and can be considered as an independent factor of survival (111, 112). In comparison, there was no decreased survival in case of SGZ involvement or corpus callosum invasion (111). Importantly, the proximity with the SVZ does not allow assessing the origin of GBM. Indeed, Han et al. reported that GSCs display similar stem gene expression in GBM with and without LV contact (113). In the same way, Mistry et al. reported that SVZ contact is not associated with molecular signatures in GBM bulk tumor (114). Finally, in the latest study by Comas et al*.*, an analysis of GBM progression in 133 adult patients with primary GBM treated with the standard TMZ-based adjuvant radiochemotherapy showed that GBM in contact with the SVZ appears to be an independent prognostic factor for poorer progression free-survival (PFS) but not for OS. They also reported that SVZ-contacting tumors were associated with a higher rate of contralateral relapses and more aggressive recurrences which they defined as relapses occurring in patients presenting a sudden worsening of their clinical condition before it could be detected by the follow up MRIs taken every 3 months. They concluded that a direct contact of GBM tumors with the SVZ could be used as a prognostic factor (115).

A similar retrospective analysis was recently conducted in 63 children and adolescents (median age of 12.3 years) diagnosed with HGG (116). Tumors contacting the SVZ were found in 54% of the patients and were usually larger than tumors not in contact with the SVZ. Furthermore, patients with SVZ-associated tumors had a decreased survival time (HR = 1.94, 95% CI 1.03–3.64, *p* = 0.04). Thus, similarly to adult findings, these data suggest that in children and adolescents, the presence of HGG attached to the SVZ is associated with a poorer prognosis (116). Targeting the SVZ could therefore be a common therapeutic target for adult GBM and pediatric HGG.

### A Potential Therapeutic Target

#### Surgery

While more and more studies have highlighted the importance of Gross Total Resection, nay Supratotal Resection in regard to OS (117, 118), it remains unclear how large the resection should be when a GBM tumor touches the SVZ. Some surgeons are indeed reluctant to open the ventricle in order to obtain a complete resection of the tumor, as it has been associated with communicating hydrocephalus or tumor spread among the ependyma or *via* the cerebro-spinal fluid (119). However, in a retrospective study of 229 GBM adult patients, Behling et al. showed that ventricular opening was not associated with a reduced OS in a multivariate analysis and could therefore be considered to achieve gross total resection (120). Moreover, Saito et al. recently performed a retrospective study with 111 GBM adult patients and reported that a wide ventricle opening (>23.2 mm) is a strong predictive factor for longer OS (121). Information on surgical intervention for pediatric glioma tumors contacting the SVZ is lacking. Thus, retrospective and prospective studies are undeniably needed to confirm those results in children.

#### Irradiation

As the SVZ involvement worsens the prognosis, it seems sensible to find a way to interfere with it. In this context, many studies have considered the specific ipsilateral, nay bilateral irradiation of the SVZ, even in absence of neuroradiological clues of the presence of tumoral cells in this brain region. In 2016, Smith et al. reviewed the different studies that investigated the advantages of irradiating the SVZ to improve the OS for adult GBM patients (122). We updated their findings to include the latest publications on that topic (**Table 1**). Evers et al. published the first retrospective study revealing an improvement of PFS after bilateral irradiation of the SVZ with a median dose superior to 43 Gy (PFS: 15.0 vs 7.2 months, *p* = 0.028) for patients suffering from grade III/IV gliomas (123). In a similar way, another retrospective study of 40 patients reported a better OS if a dose equal or superior to 53.6 Gy was delivered to the ipsilateral SVZ (iSVZ) (124). Inversely, Slotman et al. used the same cut-off of 43 Gy for bilateral SVZ irradiation and did not observe any difference in OS or PFS in their retrospective study. However, and importantly, they reported less distant recurrences in case of a delivered dose greater than 43 Gy to the contralateral ventricle (131). However, the conclusions of those three studies suffer from a limited number of patients (55, 40, and 40, respectively) (123, 124, 131).

**Table 1:** Summary of the advantages of irradiating the subventricular zone to improve the overall survival for adult glioblastoma (GBM) patients.

Authors (year)	Study design	Number of patients (tumor subtype)	Median delivered dose	Outcomes	
*Studies in favor*					
				PFS	OS
Evers et al. (123)	Retrospective	55 (Gliomas grade III/IV)	> 43 Gy to biSVZ	15 *vs* 7.2 months (*p* = 0.028)	
Gupta et al. (124)	Retrospective	40 (GBM)	>53.6 Gy to cSVZ and iSVZ		Improved on multivariate analysis in group with >53.6 Gy to iSVZ
Lee et al. (125)	Retrospective	173 (GBM)	>59.4 Gy to iSVZ	Improved on univariate and multivariate analysis	
Chen et al. (126)	Retrospective	116 (GBM)	>40 Gy to iSVZ after GTR	15.1 months *vs* 10.3 months (*p* = 0.028)	17.5 months *vs* 15.6 months
Iuchi et al. (127)	Prospective	46 (GBM)	>50–60 Gy to SVZ		36.2 months in patients with SVZ necrosis *vs* 13.3 months in patients without SVZ necrosis
Ravind et al. (128)*	Retrospective	50 (GBM)	>50 Gy to iSVZ > 37 Gy to cSVZ		Improvement for the iSVZ: 19.83 months *vs* 6.07 months (*p* = 0.031) No improvement for the cSVZ
Foro et al. (129)*	Retrospective	53 (GBM)	>52.2 Gy to iSVZ, >51 Gy to cSVZ, >47.2 Gy to biSVZ		Improvement for patients receiving >51Gy in the cSVZ
Foro Arnalot et al. (130)	Retrospective	65 (GBM)	>48.8 Gy to cSVZ		Improvement
*Studies not in favor*					
Slotman et al. (131)	Retrospective	40 (GBM)	>43 Gy to iSVZ, cSVZ, biSVZ	No correlation with iSVZ, cSVZ, or biSVZ dose	No correlation with iSVZ, cSVZ, or biSVZ dose
Elicin et al. (132)	Retrospective	60 (GBM)	>59.2 Gy to cSVZ	7.1 months *vs* 10.37 months	
Anker et al. (133)*	Retrospective	88 (GBM)	>56.4 Gy to iSVZ, >33.4 Gy to cSVZ	No correlation with iSVZ, cSVZ, or biSVZ dose	No correlation with iSVZ, cSVZ, or biSVZ dose
Sakuramachi et al. (134)*	Retrospective	54 (Gliomas grade III/IV)	>58.2 Gy to iSVZ, >44.1 *Gy* to cSVZ	No correlation with iSVZ, cSVZ, or biSVZ dose	No correlation with iSVZ, cSVZ, or biSVZ dose
Murchison S et al. (135)	Retrospective	370 (GBM)	>59.4 Gy to iSVZ, cSVZ or biSVZ	No correlation with iSVZ, cSVZ, or biSVZ dose	No correlation with iSVZ, cSVZ, or biSVZ dose
Valiyaveettil et al. (136)	Retrospective	95 (Gliomas grade III)	>54 Gy to iSVZ	Decreased in univariate analysis, No correlation in multivariate analysis	
Valiyaveettil et al. (137)	Prospective	74 patients (GBM)	>50 Gy to iSVZ	No correlation with iSVZ, cSVZ, or biSVZ dose	No correlation with iSVZ, cSVZ, or biSVZ dose

*Some retrospective studies presented in meetings reported opposite results, in favor (128, 129) or not (133, 134) in favor for SVZ irradiation. This table is an update of Smith et al. findings and includes the latest publications on that topic (122).

Later, using a larger cohort of 173 patients, Lee et al. retrospectively showed an increased PFS for an ipsilateral SVZ irradiation with a delivered dose superior to 59.4 Gy (125) Interestingly, Chen et al. showed that an increased iSVZ irradiation (superior or equal to 40 Gy) after GTR was associated to a better PFS and OS (126). Another retrospective study showed a poor PFS if the dose delivered to the contro-lateral SVZ (cSVZ) was superior to 59.2Gy (132). Those studies are in fact rather difficult to compare as they are retrospective studies and do not control for important variables such as (i) patient selection, (ii) irradiation dose, (iii) cut-off values, or (iv) importantly, the delineation of the SVZ. Moreover, classical prognostic factors such as MGMT or IDH status have frequently not been considered. Besides, a prospective study initially designed to test hypofractionated high-dose intensity modulated radiation therapy reported a better OS in case of radionecrosis in the SVZ (127).

In 2017, Foro Arnalot et al. reported another retrospective study of 65 patients showing an improvement in PFS but not in OS if the cSVZ received a dose superior or equal to 48.8 Gy (130). Khalifa et al. showed that a dose inferior to 20 Gy for bilateral SVZ irradiation was associated with poor prognosis (138). In 2018, Murchison et al. did not found any correlation between SVZ dose and PFS/OS in a large retrospective study of 370 GBM patients (135). Recently, in a retrospective study of 95 patients suffering from anaplastic gliomas, Valiyaveettil et al. reported no correlation between SVZ dose and PFS/OS in multivariate analysis (136). In a prospective study including 74 GBM patients, the same team did not found any correlation between SVZ dose and PFS/OS (137). Finally, in a short prospective study of 30 GBM patients, the sparing irradiation of neurogenic niches, including SVZ, did not modify PFS or OS in comparison to a matched historical control (139).

In this context, a phase II clinical trial combining standard radio- and chemotherapy to deliver irradiation to the ipsilateral (60 Gy) and the contralateral (46 Gy) SVZ is ongoing (NCT02177578). This study will provide valuable information on benefits that targeting the SVZ could offer for the treatment of GBM. It has to be said that the major constraint to investigate SVZ irradiation more deeply, or even to consider SGZ irradiation, is the fear that it might hasten neurocognitive decline as healthy NSCs would not be spared by the treatment and would suffer along with tumor cells. While some of the cited studies did not show a correlation between the delivered irradiation dose to SVZ and changes in performance status, Iuchi et al. found that high dose radiations deliver to the SVZ, leads to radionecrosis and better OS. However, it also results in progressive decline in Karnofsky performance status which measures the ability for a patient to carry out daily tasks (127). Henceforth, it is not surprising that some clinical trials focused on sparing the SVZ and the hippocampus.

In children, retrospective and prospective studies suggest an association between neurocognitive deficits and radiation dose to the hippocampus hosting the SGZ, but not the SVZ. Due to the rarity of HGG in children, the link between SVZ irradiation and survival has not yet been investigated (126, 140–143).

### Targeting GSCs Nested in the SVZ

Many current researches aim at targeting GSCs. To do so in an efficient manner, it is important to know what to target. The most common pathways involved in GSC maintenance include the Wnt, the Sonic hedgehog (SHH) and the Notch pathways. As recently discussed by Sharifzad et al., targeting these pathways could help eradicating GSCs or increase chemotherapy efficiency (144). To target GSCs nested in the SVZ, it has been suggested to use perphenazine, an inhibitor of the dopamine receptor D3, in order to block the migration of GBM cells to the SVZ (145). As the activation of the dopamine receptor D3 in SVZ cells is associated with their proliferation *in vitro* (146), its blockade by perphenazine could also maintain GSC in a quiescent state in the SVZ. Bardella et al. reviewed another interesting approach which consists of interfering with the SVZ inflammatory environment as it might predispose cells to mutations and worsen cancer phenotypes (147). However, while it has been largely reported that microglia participate in GBM progression locally by adopting an anti-inflammatory state (148), their interaction and effects on SVZ-nested GSCs remain to be proven.

## Conclusion

HGG account for a high proportion of death resulting from cancer, both in adults and children. Unfortunately, survival has not been significantly improved over the last decades. Both bench and bedside evidences strongly support the involvement of GSCs and SVZ in HGG recurrences. We and others, previously demonstrated that GSCs migrate from the tumor mass toward the SVZ, through the CXCL12/CXCR4 axis or through a pleiotrophin-driven axis. Once hosted in the SVZ, GSCs benefit from a protective environment providing increased resistance to irradiation and chemotherapy, before these cells get reactivated by a still unknown mechanism and recolonize the TM or invade other sites. In adults, the benefit/risk balance of targeting the SVZ by surgery and/or radiotherapy was investigated in clinical settings; however, the review of the current literature does not permit a clear conclusion yet. In children, it has not been evaluated and should be further investigated. Other technical approaches to target the SVZ also remain to be explored. Blocking the migration of GSCs toward the SVZ is probably not an option, given that the cells would already have migrated out at the time of the diagnosis. Other possibilities could be to decrease or to block the recolonization of the TM. The cancer cell trap approach is another interesting and original concept that exploits the migratory potential of cancer cells in order to concentrate them toward specific locations (149). This approach has been showed to reduce the metastatic potential of human breast cancer cells implanted in female mice, through biomaterial scaffolds implanted in peritoneal fat pads (150, 151). This kind of approach should definitely be further investigated in the context of pediatric and adult HGG and DMG and could be combined with local targeted therapies.

In conclusion, there is strong evidence that the migration of GSCs toward the SVZ is implicated in HGG recurrences, both in adults and in children. The exact mechanisms supporting this process should be further investigated with the perspective of specifically targeting this particular cell population.

## Author Contributions

AL, MD, and CD performed the literature review, and wrote the manuscript. BR edited the manuscript. CP and NC conceived, supervised, and edited the manuscript. All authors contributed to the article and approved the submitted version.

## Funding

AL received the Clinical Researcher of the FNRS-Belgium. MD is a PhD student funded by the TELEVIE-FNRS. CD is PhD student supported by the EUROMA funds (Fondation Léon Frédéricq, University of Liège). NC is supported by the Neurological Foundation of New Zealand (Philip Wrightson Postdoctoral Fellowship). BR research group is supported by the FNRS, the University of Liège and the Fonds Léon Frédéricq.

## Conflict of Interest

The authors declare that the research was conducted in the absence of any commercial or financial relationships that could be construed as a potential conflict of interest.
